# Is the process of successful sexual aging different in older partnered and non-partnered adults?

**DOI:** 10.1371/journal.pone.0344655

**Published:** 2026-03-13

**Authors:** Aleksandar Štulhofer, Azra Tafro, Laura Pietras, Ivan Landripet, Goran Koletić

**Affiliations:** 1 Faculty of Humanities and Social Sciences, University of Zagreb, Zagreb, Croatia; 2 Faculty of Forestry and Wood Technology, University of Zagreb, Zagreb, Croatia; 3 Institute for Sex Research, Sexual Medicine and Forensic Psychiatry, University Medical Center Hamburg-Eppendorf, Hamburg, Germany; 4 Junior Research Center for Sexual and Reproductive Health in Overweight and Obesity, University Medical Center Hamburg-Eppendorf, Hamburg, Germany; University of Glasgow, UNITED KINGDOM OF GREAT BRITAIN AND NORTHERN IRELAND

## Abstract

As populations in the Western world age, interest in the sexual health and well-being of older adults has grown substantially, yet positive models of sexual aging remain underexplored—especially across diverse relational contexts. To address this gap, the Successful Sexual Aging (SSA) model was recently proposed. This model conceptualizes positive sexual aging as a dynamic process involving three interrelated psychosocial domains: acceptance of physical and sexual changes, adaptation to those changes, and the presence of opportunities for sexual expression. These domains were operationalized as a latent construct, which was validated and explored for culture-specific elements in partnered older adults. Given the importance of a close partnership for older adults’ sexuality, but also the fact that many older adults, women in particular, are non-partnered, the current study focused on the assessment of differences in positive sexual aging between partnered and non-partnered older German (*N* = 1,328, *M*_age_ = 69.2) and Croatian adults (*N* = 301, *M*_age_ = 68.8). Using measurement invariance testing and a network analytic approach, we identified important differences in reported obstacles to sexual expression between partnered and non-partnered older adults, and the robustness (connectivity) of the network of SSA-indicating items. Overall, SSA seemed to be somewhat more difficult for older non-partnered adults compared to their partnered peers. Although the 9-item measure of SSA can be used to reliably assess the process of sexual aging in both partnered and non-partnered older individuals, direct comparisons should be avoided due to partnership-specific properties of positive sexual aging.

## Introduction

With the Western population aging, there is a growing recognition of the importance of sexual health and wellbeing in later life [1–4]. However, positive frameworks of sexual aging, particularly those sensitive to relationship status, remain underdeveloped. To address this, the conceptual model of Successful Sexual Aging (SSA) [5], was recently introduced as a non-normative, developmental conceptualization of positive sexual aging [6]. The non-normative character of SSA rests on the assumption that the absence of distress about sexual inactivity—rather than the presence of sexual activity—is the prerequisite for positive sexual aging. The SSA identifies three interrelated psychosocial processes: (1) acceptance of physical and sexual changes, (2) adaptation to those changes, and (3) changing opportunities for sexual expression. The conceptual model also proposes a set of micro, meso, and macro moderators and correlates of the three processes [5]. Conceptually, the model aspires to universal validity, although intrapersonal (e.g., health-related difficulties), interpersonal (e.g., not having a partner or a partner’s lack of interest), and sociocultural obstacles (e.g., economic constraints, restrictive social norms such as sexual ageism) have been noted (5). SSA is not automatically ruled out for non-partnered older adults. Not having a partner does not preclude solitary sexual expression, nor is sexual activity in later life limited solely to long-term relationships.

Efforts to operationalize SSA resulted in the publication of the Successful Sexual Aging Scale (SSAS), the nine-item 3-dimensional measure that was initially validated in two European countries, Croatia and Germany [5]. While preliminary findings confirmed the measure’s reliability across age, gender, and culture, as well as associations with indicators like self-assessed health, subjective age, positive attitudes about sexuality in older age, relationship satisfaction, and life satisfaction in the two samples of older partnered adults (mean age was 71.2 years in the Croatian and 69.2 years in the German samples), less is known about how SSAS operates in non-partnered older adults—a group often overlooked despite its size, especially among women [6]. Initial findings from the German sample suggested a satisfactory fit of the measure when tested in non-partnered older women and men (*M*_age_ = 69.2) but, given that the Croatian sample did not include non-artnered older adults, no attempt was made to explore measurement invariance of the SSAS across partnership status. Therefore, little is understood about how relationship status might shape the internal structure of SSAS across different cultural contexts. Although the need for further research on cross-cultural aspects of sexual aging has been recognized [7,8], this study used cultural heterogeneity not for comparative purposes, but for a methodological one—to address, in a robust manner, the gap in understanding the role of relationship in positive sexual aging. To that end, we applied a network analytic approach to compare SSAS patterns among partnered and non-partnered older adults in Croatia and Germany—two countries with distinct sociocultural and socioeconomic recent histories, particularly relevant for older cohorts—to contribute to the emerging literature on positive sexual aging [9–12].

### Relationship status, gender, and sexual aging

Partnership status plays a crucial role in shaping sexual and emotional well-being in older adulthood. Being married or in a long-term relationship has been consistently linked to a reduced mortality risk [13] and greater emotional support, particularly important due to aging-related reduction of social ties [14,15]. These insights are also highly relevant in the context of sexual aging. Multiple studies have found that partnered older adults tend to report greater sexual activity and satisfaction compared to non-partnered individuals [16,17]. This is especially evident in older women, who tend to outlive their male partners [2,18,19].

A large-scale longitudinal Dutch study found that older adults without a partner, especially women, perceived sex as less important than their partnered peers [20], though the value placed on sex has increased across recent generations, particularly among non-partnered women. In a cross-national study of four European countries, partnered individuals were more likely to report sexual activity and satisfaction, as well as higher sexual wellbeing [11,21]. Similarly, national data from the UK and Germany showed significantly higher sexual activity and satisfaction among partnered individuals [16,22].

Despite the evidence of a moderating role of relationship status in sexual activity, motivation, and sexual satisfaction among older adults from different countries, the mechanisms through which relationships impact sexual aging are not well understood. A few international qualitative and mixed-method studies suggest that emotionally supportive relationships enable adaption and continued sexual expression and sexual well-being in older age by fostering erotic flexibility (i.e., de-centering intercourse as the primary component of sexual activity) and mutual encouragement in adjusting to the changes in sexual expression that come with aging [23,24]. These findings align with a broader framework of successful aging that emphasize emotional connectedness, adaptability, and interpersonal support [25].

Importantly, (perceived) relationship quality is likely of central importance. Studies show that emotional intimacy, mutual satisfaction, and sexual communication are closely tied to sexual well-being in older couples [26–28]. Intriguingly, there is some evidence that re-partnering in later life may even enhance sexual activity, likely due to novelty and renewed erotic interest [29], which suggests that in at least some cases long-term relationships can negatively affect partners’ sexuality [30].

Gender disparities in how relationship status relates to sexual aging are plausible, but evidence is sparse. Given the established gender differences in self-reported health, well-being, and successful aging [31], unequal division of household labor, and differential costs of discordant marriage [32], more attention should be paid to this issue. Qualitative research on sexual aging found that female sexual aging is often negatively influenced by traditional gender norms [33] and marital power dynamics [34,35]. Moreover, a dyadic study observed that male partners’ levels of emotional intimacy were significantly associated with their female partners’ sexual well-being scores, whereas female partners’ emotional intimacy was unrelated to their male partners’ sexual well-being—highlighting gender asymmetries in emotional and sexual interdependence [28].

### Study aims

To address the current lack of evidence about the role of relationship status in positive sexual aging, the aim of the current study was to assess potential differences in SSA between older partnered and non-partnered adults in two countries (Croatia and Germany) using measurement invariance testing and network analytic approach. While the former explores whether a scale (here, a 9-item measure of successful sexual aging) measures the same construct, and in the same manner, across older partnered and non-partnered adults [36], the latter approach compares the structure of relationships (i.e., edges) between the items (i.e., nodes) across the two groups [37]. Building on the findings from a recent study that explored the structure of SSA cross-culturally [38], we focused our analyses on testing two hypotheses:

Hypothesis 1 (H1): Due to potential benefits to sexual aging provided by a steady partner, partnered and non-partnered older adults will understand and respond to nine SSA items differently--resulting in measurement non-invariance across the two groups.

Hypothesis 2 (H2): The SSA model assumes that the three underlying psychosocial processes are interrelated [6]. Given the importance of relationship status for older adults’ opportunities for sexual activity, as well as for their acceptance and adaptation to aging-related changes in sexuality, the global structure of SSA network (represented by associations between nine items) is hypothesized to differ between partnered and non-partnered older adults. Because partners likely play a role in facilitating acceptance and adaptation, as well as reducing obstacles to sexual activity, the network’s density (the number of associations among SSAS items) and its connectivity (the structure that holds the network together) are expected to be higher—i.e., denser and more strongly connected—among partnered compared with non-partnered participants, reflecting the partner’s influence.

## Method

### Participants and procedure

#### German sample.

In the late 2024, 1,328 older German adults (65 + years; *M*_age_ = 69.2, *SD* = 2.98) were included in a larger national study focused on sexual and reproductive health. The study participants, adults aged 18–75 years, were recruited through an online panel established by a German social research institute (overall response rate was 13.0%). A quota sampling based on age, gender, education, and regional distribution was applied to reflect the composition of the German online population. After receiving information about the study, electronic informed consent was obtained prior to participation, with individuals required to actively indicate their agreement by selecting an online consent option before accessing the survey. Median time to complete the online questionnaire was 36 minutes. Ethical approval for the study (LPEK-0814) was obtained from the local Psychological Ethics Committee (SCREENED FOR REVIEW).

#### Croatian sample.

In May 2025, 301 older partnered and non-partnered adults aged 65 + years (*M*_age_ = 68.8, *SD* = 4.49), who were members of a national quota-based commercial online panel, completed a brief online questionnaire on sexual aging. Planned sample size (*N* = 300) was determined by limited study funding. The response rate was 38%; average time to complete the questionnaire was under 10 minutes. Informed consent was obtained electronically. Before accessing the survey, participants indicated their consent to participate by actively selecting an online agreement option. The study procedures were approved by the Research Ethics Committee of the (SCREENED FOR REVIEW).

### Measures

Together with gender, age, and relationship status (dichotomized into 1 = single and 2 = married or in a relationship), the SSAS was used to measure the process of positive sexual aging [5]. The measure was developed in Croatian and English, and then translated into German using the standard double translation procedure with two independent translators. The scale represents three dimension: (1) *acceptance* of aging-related changes in physical appearance and sexual function (e.g., “At my age, I still try to look good.”); (2) *adaptation* to aging-related changes in physical appearance and sexual function (“At my age, I can still enjoy sex”); and (3) *opportunities* for sexual expression (e.g., “Current circumstances in which I live…”)—each represented by three items. Responses were anchored with a 5-item Likert-like scale (1 = it does not relate to me at all, 5 = it completely relates to me). One of the three opportunities items was formulated differently for partnered and non-partnered participants. For the former, the item read: “My partner’s negative reactions”, while “Difficulties in finding a suitable partner” was the version for the latter. For higher scores to denote higher SSA, the three opportunities items were reverse-recoded.

### Analytical approach

Using multigroup confirmatory factor analysis (CFA), measurement invariance analysis was carried out only in the larger German sample, because of the Croatian sample’s insufficient power when divided into two groups. Network analysis was first employed in the German and then repeated in the smaller, Croatian, sample. Measurement invariance of the SSAS across older partnered and non-partnered German adults was carried out by comparing three models—configural (factor structure is assumed to be equal across the two groups), metric (equality of factor loadings is assumed), and scalar invariance (equality of item intercepts is assumed)—with increasingly constrained parameters (van de Schoot et al., 2012). Differences between the models were assessed using changes in comparative fit index (CFI) and root mean square error of approximation (RMSEA), with differences ≤ 0.01 and ≤ 0.015 between a less and a more constrained models indicating, respectively, that the two models fit the data equally well [39].

To enable more detailed insights into the latent construct of SSA, an exploration of the structure of the SSAS across partnered and non-partnered older adults was carried out by country using a network analytic approach [40]. The nine SSAS items represented network *nodes* and conditional associations (i.e., partial Spearman rank-correlations) between them represented *edges* [37]. A regularized network was obtained using EBICglasso estimator with the default regularization parameter (0.5). Missing data was excluded pairwise (<2% of data was missing). To assess the robustness of the obtained estimates, we followed the recommended methodology [41,42]. Generally, the network can be estimated on random subsamples, and similarity of the results to the original results indicates a reliable estimate. For edge weights, each sample was split randomly in two halves, weights were estimated separately, and the results were averaged. For the network centrality measures, we applied the case-dropping bootstrap approach, where the network is estimated on a decreasing subsample. Each time, the correlation between the new and the original centrality indices is calculated. In most cases, a correlation of 0.7 or above is considered acceptable, indicating a stable estimate. Both the split-halves and the case-dropping process were repeated 1000 times and we reported mean values and the corresponding 95% confidence intervals. Detailed results are provided in the supplementary file. Centrality refers to various measures that identify nodes whose positions are especially important for the overall connectivity of a network [43].

Due to the observed node clustering in the SSA network (i.e., three distinct factors), we employed participation coefficient, a bridge centrality measure, and global strength impact measures, as well as minimum spanning tree analysis [41], to describe the structure of the network by country. While participation coefficient reflects both the number and strength of a node’s connections (i.e., edges) to nodes from other clusters [41], global strength impact assesses a node’s importance for global network connectivity [44]. In other words, global strength impact reflects how the presence (or absence) of a node alters the overall connectedness of the network.

Finally, a minimum spanning tree is a method that minimizes network connections to create a „skeleton structure“ with only the essential nodes and edges retained. The pivotal node in such reduced network topology is less sensitive to small sample sizes than other centrality indices [41].

To directly compare the SSA network in partnered vs. non-partnered older adults we used the Network Comparison Test [45]. In this case, the NCT entailed two global tests: network structure and global strength invariance. While the first tests if the overall topology (i.e., the composition of edges) is statistically indistinct across two groups, the second explores whether differences in global network connectivity (i.e., weighted absolute sum of all edges) across two groups are non-significant.

In addition to a relatively high homogeneity of nodes in the SSA network (the three clusters) [46], another reason for not reporting the standard centrality indices (i.e., strength, closeness, and betweenness) was the well-documented instability of network estimations in smaller samples [37,42]. Given the known instability of network estimations in smaller samples [47], the findings from the Croatian sample should be interpreted with caution and considered meaningful to the extent that they mirror the network patterns observed in the German sample.

For statistical analyses, we used JASP 0.19.3 SEM module [48] and R version 4.4.1 [49] with ‘qgraph’ [50], ‘igraph’ [51], ‘bootnet’ [42], and ‘NetworkComparisonTest’ [45] packages.

## Results

### Sample characteristics

About two thirds (66.9%) of participants were partnered in the German sample (40.1% of female participants). The proportion was similar (70.4%) in the Croatian sample, which included more female participants (50.8%). Average length of relationships was 33.5 years (*SD* = 15.4) in the German and 37.5 (*SD* = 14.7) in the Croatian sample. Participants with a college or higher education were clearly overrepresented in Croatia (50.4% of the sample), but not Germany (29.5%). Good health was reported by 53.7% of German and 41.8% of Croatian older adults, while poor health characterized 11.4% and 13.0% of participants, respectively. Sexual activity in the past 12 months, which was defined more broadly in Croatia (“sexual intercourse or any other erotic activity”) than in Germany (“sexual intercourse”), was reported by 74.4% and 47.0% of participants in the two countries. The substantially different prevalence seems to reflect distinct operationalizations.

### Measurement invariance testing

To test the H1, we explored measurement invariance of the SSAS in older partnered and non-partnered German adults using multigroup CFA. The measure was characterized by configural and metric, but not scalar invariance across the two groups (Table 1), which precluded a direct comparison of SSAS latent mean scores between partnered and non-partnered participants. As an illustration, comparisons of 95% confidence intervals around composite means for three dimensions revealed statistically significant differences (*p* < 0.05) in the adaptation and opportunities processes, with older partnered Germans scoring higher than their non-partnered peers.

**Table 1:** Measurement Invariance Testing of the Successful Sexual Aging in the German Sample.

	Configural invariance	Metric invariance	Scalar invariance
χ^2^ (df)	141.26 (46)	164.71 (52)	395.13 (58)
CFI	0.977	0.973	0.919
∆ CFI		0.004	0.066
TLI	0.964		
RMSEA (90% CI)	0.056 (0.045–0.066)	0.057 (0.047–0.067)	0.093 (0.085–0.102)
∆ RMSEA		0.001	0.044
SRMR	0.043		

Importantly, group differences in item intercepts were the largest in the case of two opportunity items. The first (SSA6) asked about partner’s negative reactions (for partnered participants) and difficulties in finding a sexual partner (for non-partnered participants). The second asked (SSA5) about negative reactions of people in the participant’s immediate surrounding. In both cases, partnered participants reported substantially lower obstacles to their sexual expression than their non-partnered peers.

### Global network characteristics

**Fig 1:**
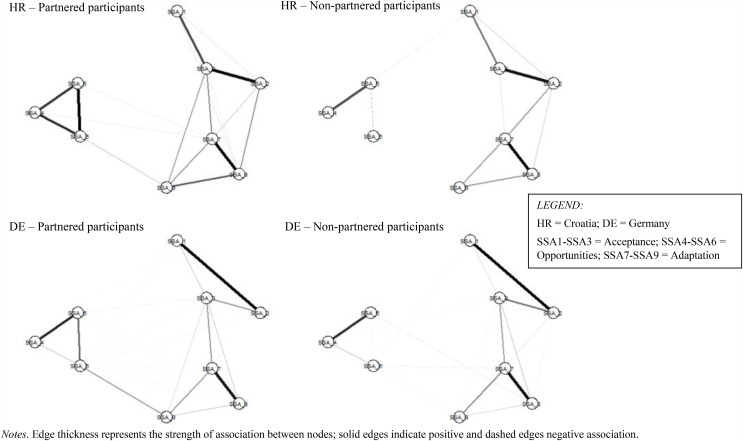
Successful Sexual Aging Network in Partnered and Non-partnered Older Adults in Croatia (HR) and Germany (DE).

Regularized country-specific SSA networks were estimated and plotted by country and relationship status (Fig 1). Following recommendations [42], information about the stability and robustness of network estimations is provided in the supplementary file. Confirming a visual inspection of the four networks, both network density (the number of non-significant edges) and average absolute edge weight (i.e., the mean partial correlation between all pairs of edges in a network) were higher in partnered than non-partnered older adults in both countries (see Table 2)—supporting the first part of H2. Additional differences were found in bridging centrality, indicated by participation coefficient. In partnered, but not non-partnered older adults, the sexual agency/adaptation node (SSA9; “I always clearly communicate to my partner what I like and dislike in sex”) was characterized by the strongest connection to acceptance and adaptation nodes in the Croatian and German samples.

**Table 2:** Global Characteristics of the Successful Sexual Aging Network in Partnered and Non-partnered Older Adults by Country.

	Croatia		Germany	
	Non-partnered participants	Partnered participants	Non-partnered participants	Partnered participants
Density	0.36	0.53	0.53	0.67
Average absolute edge weight	0.07	0.10	0.09	0.11
Global strength impact	SSA2 (0.61)	SSA2 (0.48)	SSA2 (0.99)	SSA2 (0.71)
Participation coefficient^a^	SSA6 (0.55)	SSA9 (0.57)	SSA6 (0.53)	SSA9 (0.58)
Pivotal node in minimum spanning tree	SSA8	SSA8	SSA7	SSA7

^a^Bridging centrality measure.

In both samples, perceived partner availability (SSA6; absence of “difficulties in finding a sexual partner”) was the node with the highest bridging centrality among non-partnered participants, while the agency/adaptation node (SSA9) was characterized by the highest bridging centrality in partnered participants.

**Fig 2:**
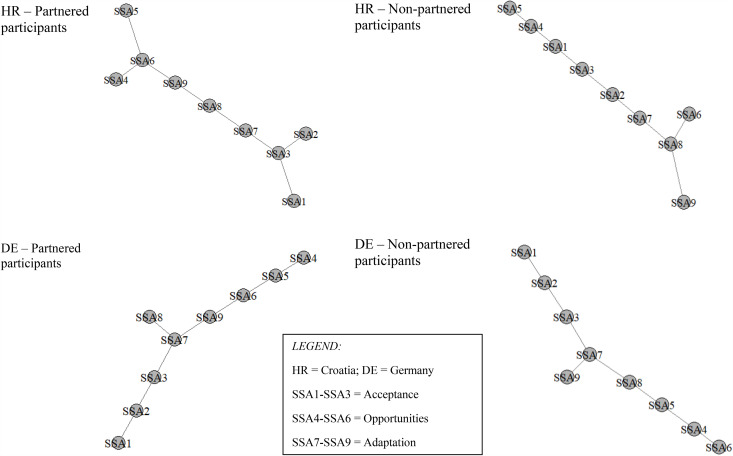
Minimum Spanning Tree (Reduced) Network in Partnered and Non-partnered Older Adults by Country.

Interestingly, we observed no differences between partnered and non-partnered participants in either country concerning the highest general strength impact node. In all four groups of participants, it was an acceptance node (SSA2; “I still find things about how I look that another person may find attractive”) that was characterized by the highest overall network connectivity. In contrast, the pivotal node in the reduced minimum spanning tree version of the SSA network was country-specific. While the first adaptation node (SSA7; “At my age, I can still enjoy sex”) occupied this central position in the German sample, the second adaptation node (SSA8; “I am content with how, at my age, my body reacts to sexual touch”) did so in the Croatian sample (see Fig 2).

Finally, connections between the three opportunities nodes, SSA4 (“life circumstances, such as privacy issues”), SSA5 (absence of “other people’s negative reactions”), and SSA6 (absence of “partner’s negative reactions to sex” [for partnered participants] and absence of “difficulties in finding a sexual partner” [for non-partnered participants]) were, overall, markedly stronger in partnered compared to non-partnered participants in both countries. In the German sample, these association were in the 0.48–0.17 range in partnered and the 0.40–0.01 range in non-partnered older adults. In the Croatian sample, the respective ranges were 0.40–0.31 and 0.19–0.01.

### Network comparisons

To test the second hypothesis (H2) more directly, two global network comparison tests were carried out: network invariance and global strength invariance tests [45]. Both tests are based on the initial assumption that network edges are invariant across the two groups of participants. The assumption was rejected in both samples, the German (*M* = 0.33, *p* < 0.001) and Croatian (*M* = 0.34, *p* = 0.03), indicating that the SSA network was group-specific. The second test, which reached significance in the German sample (S = 0.72, *p* < 0.001) and approached significance in the underpowered Croatian sample (S = 0.90, *p* = 0.06), provided direct support for the second part of H2: higher global network strength was observed among partnered compared to non-partnered participants in both countries (Germany: *M*_partnered_ = 3.81 vs. *M*_non-partnered_ = 3.09, and Croatia: *M*_partnered_ = 3.58 vs. *M*_non-partnered_ = 2.69).

## Discussion

Recently, a novel concept of positive sexual aging (the SSA) was introduced and operationalized as a 9-item latent construct [5]. Although the measure was extensively validated (it is currently being explored for cultural differences), its characteristics in partnered vs. non-partnered older adults have not been systematically explored. The current study represents an initial step in bridging this gap by exploring measurement invariance and the structure of connections between the SSAS items across older partnered and non-partnered individuals.

Before discussing this study’s findings, a possible objection needs to be tackled first. Can the SSAS be used to analyze sexuality of non-partnered older adults? Are the three core processes that constitute the SSA model relevant only (or mostly) for partnered older adults? Although partially different, primarily in the sense that sexual partner availability is a more challenging obstacle to sexual activity for non-partnered compared to partnered older adults, the opportunities dimension seems highly relevant for both groups of older adults. Privacy issues, as well as disapproving or judgmental social environment, may also be problematic for non-partnered older women or men who are looking for a sexual partner, compared to married older adults or those in a long-term relationship. Similarly, there is no reason to hypothesize that the acceptance dimension is substantially different across partnered and non-partnered older adults. Whether partnered or not, older individuals cannot avoid changes that will affect their sexuality and physical appearance. Having a long-term partner, however, especially if the partnership is characterized with high emotional intimacy, may facilitate the acceptance through partner’s support. In contrast, an overly critical and demanding partner can create difficulties in dealing with aging-related changes. Finally, the process of adaptation to aging-related changes can also greatly benefit from partners’ mutual understanding, communication, familiarity, and emotional closeness, but its constitutive components (i.e., selection, optimization, and compensation [6,52]) are equally relevant for older non-partnered adults.

There is little doubt that our measure is ill-suited for the assessment of positive sexual aging in older women and men who are either asexual or avoiding any kind of sexual experience. This, however, should not be extended to older adults who have decided, for whichever reasons, to remain sexually expressive exclusively through sexual fantasies and/or self-pleasuring. Of the nine items indicating SSA, only one (the sexual agency item) explicitly refers to a sexual partner [5]. Even for older individuals who no longer desire sexual experiences, the SSAS may still be valuable—particularly when paired with a measure of distress related to one’s sexuality. A combination of moderate SSAS scores and low or absent distress could indicate SSA in older women and men who are no longer interested in sex. Although it is not possible to quantify “moderate scores” at this stage of research into SSA, we would expect that older individuals whose disengagement from sex life is not accompanied by distress would score substantially higher than their distressed peers on all three dimensions, particularly on adaptation and opportunities. Compared to sexually active older adults, these disengaged individuals would likely differ primarily in acceptance levels.

Based on our conceptualization of positive sexual aging [5], we tested two hypotheses: that measurement non-invariance would be observed when testing the SSAS across partnered and non-partnered older adults (H1), and that the former group would be characterized by stronger interconnectedness between the three SSA processes compared to the latter group (H2). Because the size of the Croatian sample precluded multigroup testing, the first hypothesis was tested, and confirmed, only in the German sample. Although the overall latent structure of the construct was similar across partnered and non-partnered participants (i.e., metric invariance was obtained), scalar invariance—the precondition for direct comparisons between the two groups participants—was not obtained. The primary reasons for this were markedly higher obstacles to sexual expression reported by older non-partnered adults, which is consistent with reports that older women, who tend to outlive their partners, are less likely to be sexually active than older men [16,53,54].

The apparent advantage that partnered older adults have over their non-partnered peers in terms of sexual aging—consistent with existing literature on aging and sexuality [2,8,55], should not be reduced to mere partner availability. The relation between partnership and sexual aging is primarily about the quality of partners’ interactions, communication, and emotional connectedness. This is what often generates motivation, mutual encouragement, and support needed to deal with inevitable changes in physical appearance, sexual interest, and sexual performance [56–58]. An additional advantage that a close relationship may offer to aging partners is a sexually flexible environment, in which aging-related changes in sexuality are openly discussed, negotiated, experimented with, and embraced. For such relationships, a history of mutually satisfying sexual experiences clearly matters. In contrast, less connected couples (particularly those with a more problematic sexual history) may muster little or no emotional and cognitive resources needed for successful adjustment to changes, resulting in a higher risk of sexual withdrawal. Furthermore, new relationships started at older age can also be beneficial for SSA. Although little is known about the effect of familiarity on sexual interest and activity in older adulthood, it is safe to assume the phenomenon remains relevant, even if less pronounced (or distressing) than in midlife. For older women and men, particularly those who have engaged in accepting and adapting to aging-related changes to their sexuality, re-partnering, and the psychological and erotic novelty it brings [59], is likely to further enhance the process of SSA.

Our second hypothesis (H2) posited that a network representing psychosocial processes underlying SSA would be denser and better connected in partnered than non-partnered older adults. We expected that the network topology of SSA and its three clusters of nodes (each representing a psychosocial mechanism underlying the SSA model) would be interrelated more strongly among older partnered adults due to the relationship effect (i.e., partner’s support). The effect was hypothesized to manifest in stronger connections between acceptance and adaptation, which were also expected to influence, positively, (the perception of) opportunities for sexual expression. The greater connectedness of SSA network observed in partnered older adults suggests that their sexual aging may be more sensitive to changes, including those in their partner, in specific domains. For example, a decline in self-confidence regarding sexual competence or in acceptance of one’s appearance could influence other parts of the network, thereby affecting the overall process of sexual aging.

The finding that the structure of SSA was more robust in partnered compared to non-partnered participants, confirming the second hypothesis, is likely to raise questions about the association between relationship length and SSA scores. Given that the current study did not set to answer the question, the evidence about a curvilinear relationship between relationship duration on the one hand and perceived quality of sex and sexual satisfaction on the other hand [30,60] is highly relevant. In the light of increasing health adversities related to the “fourth age” [61–63], SSA should be understood as a trajectory, which will, at one point, begin to move downwards. With this in mind, disentangling the effect of relationship duration from those of partners’ aging may prove challenging.

The stronger connections between the three opportunities nodes observed in partnered participants, compared to their non-partnered counterparts, point to higher stability and predictability of the structure of sexual opportunities. An alternative interpretation of the weaker interconnectedness would be that it is simply the consequence of not having a sexual partner. Non-partnered older adults do not need to be concerned about privacy issues and negative reactions from people around them. This logic may be valid for older individuals who experience no desire for partnered sexual activity, but not for other older adults. Searching for a sexual partner is likely to be more problematic in this context than being sexually active with a steady partner or a spouse—as indicated in testing the SSAS measurement invariance. It should be noted that the opportunities items were preceded by the following text: “To what extent are the following things obstacles to your being sexually active as you would like? If you currently feel no need for sexual expression, please provide your answers imagining a situation in which you would like to experience something sexual.”

It is also important to discuss findings that did not discriminate between the two groups of participants. In the larger German sample, we observed that an acceptance node (“I still find things about how I look that another person may find attractive”) was characterized by the highest overall network connectivity, while an adaption node (“At my age, I can still enjoy sex”) occupied the central position in the trimmed down network version. This was the case among both the partnered and non-partnered participants’ SSA network, indicating that structural differences in SSA across these two groups were limited. With the caveat of relatively low replicability of network estimations [47], the first finding cautiously suggest that some level of acceptance of aging-related changes in one’s sexuality is necessary for adapting to the changes—as well as for recognizing (and even creating) opportunities for sexual expression—regardless of whether the person is partnered. Similarly, the finding that an adaptation node held together the pruned SSA network implies that the process of adaptation, while likely supported by a partner, is not determined by relationship status.

In our view, the current study contributes to the body of literature in three ways. It adds to growing evidence about positive sexual aging and its underlying psychosocial mechanisms by highlighting the importance of relationships status as one of essential moderators [6]. Secondly, the network analytic approach provided a distinct empirical perspective supportive of earlier findings about the advantages of emotionally close relationships in the context of sexual aging [22,35,55,64]. Finally, our findings are informative for sexuality education among older adults, as well as for care-taking professionals and clinicians who may overlook the fact that a substantial proportion of their clients remain motivated to express themselves sexually [65].

### Study limitations

Several study limitations should be briefly discussed. Firstly, the small proportion of non-partnered individuals in the Croatian sample rendered the associated network estimations highly unstable [42]. For this reason, the findings obtained in the Croatian sample should be considered relevant only when replicating (as a pattern) in the larger German sample. Secondly, our findings cannot be generalized to the respective population, because of the overrepresentation of better educated (thus, likely more sexual open and liberal) older Croatian adults, but also due to a very small response rate (suggestive of systematic selection bias) in the German sample.

Thirdly, sexual activity was operationalized differently in the two countries. In contrast to the broad definition used in the Croatian study, the German survey included a narrow specification (evident in a much lower proportion of sexually active older adults in the German, compared to Croatian, sample). However, given that being sexually active was not an inclusion criterion for our analyses, the difference was unlikely to affect the reported findings. Fourthly, with the majority of partnered participants reporting a long-term relationship, we did not explore if SSA levels varied with relationship duration. For example, it would be interesting to assess possible differences (or overlaps) in the structure of SSA between older non-partnered adults, those in long-term partnerships, and individuals who have recently re-partnered. Finally, the role of sexual orientation in SSA also remains a task for future assessments.

## Conclusions

Despite the documented increase in the proportion of older Western adults who find remaining sexually active important and a rising number of studies that explore sexual aging, there is a lack of conceptual clarity in the field, which hampers the transition from mainly descriptive to more analytical evidence about the process of sexual aging. The recently developed theoretical model of SSA, has been operationalized and tested in different cultural settings but without considering one of the essential moderators of sexual aging—relationships status. This study’s findings indicate that the structure of SSA differs in partnered compared to non-partnered older adults. In the former group, reported obstacles to sexual expression were lower and the core SSA processes better interconnected, suggesting that positive sexual aging might pose more challenges to non-partnered adults. More research, particularly qualitative, would be needed to shed light on the dynamics of positive sexual aging in this population. Finally, our findings indicate that the measure recently developed to assess SSA (the SSAS) can be reliably used to assess positive sexual aging in both partnered and non-partnered older adults, as well as its associations with other relevant constructs—albeit with an important caveat. Due to differences in the configuration of underlying processes, the SSAS should not be used to directly compare the two groups’ mean SSA levels.

## Supporting information

S1 Appendix Study measures. (PDF)

S2 Appendix Additional tables. (PDF)

S3 Appendix Croatian dataset. (XLSX)

S4 Appendix German dataset. (XLSX)
