# Supplementary material for: Is the process of successful sexual aging different in older partnered and non-partnered adults?
Source: PLoS One. 2026 Mar 13;21(3):e0344655. doi: 10.1371/journal.pone.0344655 (PMC12987439; doi:10.1371/journal.pone.0344655)
Supplement: S1 Appendix — (PDF) [file pone.0344655.s001.pdf]

Supporting information

S1 Appendix. Study measures.

Gender

What is your gender?

1 – Male

2 – Female

3 – Prefer not to answer

Age

How old are you? _____ years.

Relationship status

What is your relationship status?

1 – Married and living together with my spouse

2 – Married and not living together with my spouse

3 – In a relationship and living together with my partner

4 – In a relationship and not living together with my partner

5 – Not in a relationship

6 – Prefer not to answer

Successful Sexual Aging Scale

To what extent are the following things obstacles to your being sexually active as you would like? If you currently feel no need for sexual expression, please provide your answers imagining a situation in which you would like to experience something sexual

Current circumstances in which I live (e.g., lack of privacy).

Concerns about negative reactions of people around me.

My partner's negative reactions. / Difficulties in finding a suitable partner.

1 – It does not relate to me

2 – It relates to me a little

3 – It somewhat relates to me

4 – It relates to me a great deal

5 – It completely relates to me

To what extent do the following statements apply to you personally:

At my age, I still try to look good.

I still find things about my appearance that another person may find attractive.

Older people like me can be sexually attractive despite aging-related changes

Despite my age, I can still enjoy sex.

I am content with how, at my age, my body reacts to sexual touch.

I always clearly communicate to my partner what I like and dislike in sex.

. *1 – It does not relate to me*

2 – It relates to me a little

3 – It somewhat relates to me

4 – It relates to me a great deal

5 – It completely relates to me
