# Supplementary material for: Is the process of successful sexual aging different in older partnered and non-partnered adults?
Source: PLoS One. 2026 Mar 13;21(3):e0344655. doi: 10.1371/journal.pone.0344655 (PMC12987439; doi:10.1371/journal.pone.0344655)
Supplement: S2 Appendix — (PDF) [file pone.0344655.s002.pdf]

Table S1 - Global strength impact (GSI) and participation coefficient (PC) values for each item/node by country and relationship status

	Croatia				Germany			
	Partnered		Non-partnered		Partnered		Non-partnered	
**Variable**	**GSI**	**PC**	**GSI**	**PC**	**GSI**	**PC**	**GSI**	**PC**
SSA1	0.3220	0.2101	0.3614	0.1864	0.6904	0.0775	0.5373	0.0930
SSA2	0.4838	0.4838	0.6085	0.4838	0.7067	0.0507	0.9906	0.1831
SSA3	0.5416	0.4583	0.5240	0.1402	0.6356	0.5448	0.6440	0.5203
SSA4	0.4469	0.0913	0.3768	0.1990	0.4596	0.0994	0.4849	0.0539
SSA5	0.5279	0.0777	0.4361	0.0000	0.6649	0.0857	0.5935	0.1730
SSA6	0.5488	0.2304	0.1456	0.0000	0.6005	0.5086	0.2278	0.5291
SSA7	0.4827	0.5234	0.5743	0.3865	0.5733	0.3963	0.6430	0.4029
SSA8	0.5345	0.4043	0.5405	0.2365	0.5462	0.3525	0.8101	0.4193
SSA9	0.4533	0.5661	0.3703	0.3518	0.5584	0.5827	0.3874	0.3849

Table S2 - Stability of the edge weight estimates from the EBICglasso method

To estimate the stability, we conducted the following simulation: The dataset was randomly divided into two equal parts, and edge weights were estimated independently for each half. We then averaged the estimates for each edge. This procedure was repeated 1000 times to derive a mean estimate and a 95% “confidence interval” for each edge, defined as the range encompassing 95% of the observed values. The results for each network are given below.

CROATIA Partnered	SSA1	SSA2	SSA3	SSA4	SSA5	SSA6	SSA7	SSA8	SSA9
**SSA1**	0<br>0 - 0	0.059<br>0.035 - 0.096	0.266<br>0.239 - 0.287	0.001<br>-0.009 - 0.023	0.002<br>0 - 0.023	0.007<br>0 - 0.038	0.001<br>0 - 0.015	0.043<br>0.016 - 0.08	0.01<br>0 - 0.048
**SSA2**	0.059<br>0.035 - 0.096	0<br>0 - 0	0.376<br>0.347 - 0.399	-0.001<br>-0.023 - 0	0.001<br>0 - 0.02	-0.001<br>-0.022 - 0	0.121<br>0.101 - 0.144	0.167<br>0.142 - 0.186	0.037<br>0.011 - 0.079
**SSA3**	0.266<br>0.239 - 0.287	0.376<br>0.347 - 0.399	0<br>0 - 0	-0.004<br>-0.035 - 0	0<br>-0.01 - 0.004	0.005<br>0 - 0.033	0.189<br>0.168 - 0.207	0.055<br>0.031 - 0.093	0.132<br>0.106 - 0.152
**SSA4**	0.001<br>-0.009 - 0.023	-0.001<br>-0.023 - 0	-0.004<br>-0.035 - 0	0<br>0 - 0	0.307<br>0.284 - 0.329	0.326<br>0.3 - 0.347	0.028<br>0 - 0.064	0.002<br>0 - 0.021	0<br>-0.007 - 0.016
**SSA5**	0.002<br>0 - 0.023	0.001<br>0 - 0.02	0<br>-0.01 - 0.004	0.307<br>0.284 - 0.329	0<br>0 - 0	0.397<br>0.371 - 0.417	0.017<br>0 - 0.048	0.008<br>0 - 0.039	0.012<br>0 - 0.047
**SSA6**	0.007<br>0 - 0.038	-0.001<br>-0.022 - 0	0.005<br>0 - 0.033	0.326<br>0.3 - 0.347	0.397<br>0.371 - 0.417	0<br>0 - 0	0.011<br>0 - 0.043	0.006<br>0 - 0.035	0.086<br>0.059 - 0.11
**SSA7**	0.001<br>0 - 0.015	0.121<br>0.101 - 0.144	0.189<br>0.168 - 0.207	0.028<br>0 - 0.064	0.017<br>0 - 0.048	0.011<br>0 - 0.043	0<br>0 - 0	0.394<br>0.367 - 0.415	0.156<br>0.135 - 0.173
**SSA8**	0.043<br>0.016 - 0.08	0.167<br>0.142 - 0.186	0.055<br>0.031 - 0.093	0.002<br>0 - 0.021	0.008<br>0 - 0.039	0.006<br>0 - 0.035	0.394<br>0.367 - 0.415	0<br>0 - 0	0.234<br>0.211 - 0.255
**SSA9**	0.01<br>0 - 0.048	0.037<br>0.011 - 0.079	0.132<br>0.106 - 0.152	0<br>-0.007 - 0.016	0.012<br>0 - 0.047	0.086<br>0.059 - 0.11	0.156<br>0.135 - 0.173	0.234<br>0.211 - 0.255	0<br>0 - 0

CROATIA Non-partnered	SSA1	SSA2	SSA3	SSA4	SSA5	SSA6	SSA7	SSA8	SSA9
**SSA1**	0<br>0 - 0	0.072<br>0 - 0.145	0.154<br>0.027 - 0.241	0.016<br>0 - 0.103	0<br>0 - 0	-0.007<br>-0.075 - 0	0.004<br>0 - 0.052	0.001<br>0 - 0.025	-0.003<br>-0.053 - 0
**SSA2**	0.072<br>0 - 0.145	0<br>0 - 0	0.313<br>0.163 - 0.424	-0.001<br>-0.01 - 0	0<br>0 - 0	-0.001<br>-0.02 - 0	0.122<br>0.027 - 0.185	0.087<br>0 - 0.15	0.066<br>0 - 0.142
**SSA3**	0.154<br>0.027 - 0.241	0.313<br>0.163 - 0.424	0<br>0 - 0	0<br>0 - 0	-0.001<br>-0.011 - 0	0.004<br>0 - 0.05	0.038<br>0 - 0.097	0.002<br>0 - 0.032	0.001<br>0 - 0.003
**SSA4**	0.016<br>0 - 0.103	-0.001<br>-0.01 - 0	0<br>0 - 0	0<br>0 - 0	0.187<br>0.035 - 0.302	-0.01<br>-0.079 - 0	0<br>0 - 0	0<br>0 - 0	-0.004<br>-0.056 - 0
**SSA5**	0<br>0 - 0	0<br>0 - 0	-0.001<br>-0.011 - 0	0.187<br>0.035 - 0.302	0<br>0 - 0	-0.056<br>-0.156 - 0	0.003<br>0 - 0.038	0.002<br>0 - 0.041	0.001<br>0 - 0.016
**SSA6**	-0.007<br>-0.075 - 0	-0.001<br>-0.02 - 0	0.004<br>0 - 0.05	-0.01<br>-0.079 - 0	-0.056<br>-0.156 - 0	0<br>0 - 0	0.003<br>0 - 0.042	-0.001<br>-0.018 - 0	-0.004<br>-0.052 - 0
**SSA7**	0.004<br>0 - 0.052	0.122<br>0.027 - 0.185	0.038<br>0 - 0.097	0<br>0 - 0	0.003<br>0 - 0.038	0.003<br>0 - 0.042	0<br>0 - 0	0.355<br>0.191 - 0.476	0.12<br>0.008 - 0.183
**SSA8**	0.001<br>0 - 0.025	0.087<br>0 - 0.15	0.002<br>0 - 0.032	0<br>0 - 0	0.002<br>0 - 0.041	-0.001<br>-0.018 - 0	0.355<br>0.191 - 0.476	0<br>0 - 0	0.12<br>0 - 0.194
**SSA9**	-0.003<br>-0.053 - 0	0.066<br>0 - 0.142	0.001<br>0 - 0.003	-0.004<br>-0.056 - 0	0.001<br>0 - 0.016	-0.004<br>-0.052 - 0	0.12<br>0.008 - 0.183	0.12<br>0 - 0.194	0<br>0 - 0

GERMANY Partnered	SSA1	SSA2	SSA3	SSA4	SSA5	SSA6	SSA7	SSA8	SSA9
**SSA1**	0<br>0 - 0	0.577<br>0.566 - 0.586	0.16<br>0.151 - 0.167	0.009<br>0 - 0.024	0.001<br>0 - 0.009	0.012<br>0 - 0.029	0.003<br>0 - 0.017	0.008<br>0 - 0.025	0.003<br>0 - 0.016
**SSA2**	0.577<br>0.566 - 0.586	0<br>0 - 0	0.226<br>0.218 - 0.234	0<br>-0.006 - 0.002	0.001<br>0 - 0.009	0.012<br>0 - 0.028	0.002<br>0 - 0.014	0.006<br>0 - 0.023	0.006<br>0 - 0.023
**SSA3**	0.16<br>0.151 - 0.167	0.226<br>0.218 - 0.234	0<br>0 - 0	0<br>0 - 0.006	0.017<br>0.006 - 0.032	-0.003<br>-0.028 - 0	0.247<br>0.238 - 0.253	0.147<br>0.139 - 0.155	0.091<br>0.082 - 0.099
**SSA4**	0.009<br>0 - 0.024	0<br>-0.006 - 0.002	0<br>0 - 0.006	0<br>0 - 0	0.480<br>0.47 - 0.489	0.172<br>0.166 - 0.177	0<br>-0.003 - 0.004	0.008<br>0 - 0.023	0.02<br>0.01 - 0.038
**SSA5**	0.001<br>0 - 0.009	0.001<br>0 - 0.009	0.017<br>0.006 - 0.032	0.48<br>0.47 - 0.489	0<br>0 - 0	0.34<br>0.332 - 0.346	0.001<br>0 - 0.008	0.012<br>0 - 0.026	0.001<br>0 - 0.01
**SSA6**	0.012<br>0 - 0.029	0.012<br>0 - 0.028	-0.003<br>-0.028 - 0	0.172<br>0.166 - 0.177	0.340<br>0.332 - 0.346	0<br>0 - 0	-0.003<br>-0.025 - 0	0.009<br>0 - 0.03	0.186<br>0.174 - 0.198
**SSA7**	0.003<br>0 - 0.017	0.002<br>0 - 0.014	0.247<br>0.238 - 0.253	0<br>-0.003 - 0.004	0.001<br>0 - 0.008	-0.003<br>-0.025 - 0	0<br>0 - 0	0.517<br>0.507 - 0.525	0.22<br>0.213 - 0.228
**SSA8**	0.008<br>0 - 0.025	0.006<br>0 - 0.023	0.147<br>0.139 - 0.155	0.008<br>0 - 0.023	0.012<br>0 - 0.026	0.009<br>0 - 0.03	0.517<br>0.507 - 0.525	0<br>0 - 0	0.163<br>0.155 - 0.169
**SSA9**	0.003<br>0 - 0.016	0.006<br>0 - 0.023	0.091<br>0.082 - 0.099	0.02<br>0.01 - 0.038	0.001<br>0 - 0.01	0.186<br>0.174 - 0.198	0.22<br>0.213 - 0.228	0.163<br>0.155 - 0.169	0<br>0 - 0

GERMANY Non-partnered	SSA1	SSA2	SSA3	SSA4	SSA5	SSA6	SSA7	SSA8	SSA9
**SSA1**	0<br>0 - 0	0.513<br>0.488 - 0.536	0.09<br>0.078 - 0.099	0.001<br>0 - 0.015	-0.001<br>-0.016 - 0	-0.017<br>-0.05 - 0	0.001<br>0 - 0.013	0.003<br>0 - 0.024	0<br>0 - 0
**SSA2**	0.513<br>0.488 - 0.536	0<br>0 - 0	0.258<br>0.244 - 0.271	0.003<br>0 - 0.026	-0.001<br>-0.01 - 0	0.001<br>0 - 0.017	0.011<br>0 - 0.034	0.024<br>0.005 - 0.046	0.051<br>0.035 - 0.068
**SSA3**	0.09<br>0.078 - 0.099	0.258<br>0.244 - 0.271	0<br>0 - 0	0.007<br>0 - 0.034	0.001<br>0 - 0.017	-0.002<br>-0.023 - 0	0.208<br>0.195 - 0.22	0.154<br>0.141 - 0.165	0.031<br>0.014 - 0.06
**SSA4**	0.001<br>0 - 0.015	0.003<br>0 - 0.026	0.007<br>0 - 0.034	0<br>0 - 0	0.398<br>0.365 - 0.425	0.128<br>0.101 - 0.151	-0.002<br>-0.022 - 0	0.002<br>0 - 0.018	0.001<br>0 - 0.012
**SSA5**	-0.001<br>-0.016 - 0	-0.001<br>-0.01 - 0	0.001<br>0 - 0.017	0.398<br>0.365 - 0.425	0<br>0 - 0	0.014<br>0 - 0.046	0<br>0 - 0.004	0.025<br>0 - 0.049	0<br>0 - 0
**SSA6**	-0.017<br>-0.05 - 0	0.001<br>0 - 0.017	-0.002<br>-0.023 - 0	0.128<br>0.101 - 0.151	0.014<br>0 - 0.046	0<br>0 - 0	-0.025<br>-0.057 - 0	0<br>-0.007 - 0	0.007<br>0 - 0.048
**SSA7**	0.001<br>0 - 0.013	0.011<br>0 - 0.034	0.208<br>0.195 - 0.22	-0.002<br>-0.022 - 0	0<br>0 - 0.004	-0.025<br>-0.057 - 0	0<br>0 - 0	0.499<br>0.478 - 0.52	0.242<br>0.225 - 0.257
**SSA8**	0.003<br>0 - 0.024	0.024<br>0.005 - 0.046	0.154<br>0.141 - 0.165	0.002<br>0 - 0.018	0.025<br>0 - 0.049	0<br>-0.007 - 0	0.499<br>0.478 - 0.52	0<br>0 - 0	0.096<br>0.085 - 0.108
**SSA9**	0<br>0 - 0	0.051<br>0.035 - 0.068	0.031<br>0.014 - 0.06	0.001<br>0 - 0.012	0<br>0 - 0	0.007<br>0 - 0.048	0.242<br>0.225 - 0.257	0.096<br>0.085 - 0.108	0<br>0 - 0

Table S3 – Global strength impact stability by country and relationship status

To evaluate the stability of our centrality metrics (global strength impact and participation coefficient), we employed a modified version of the case-dropping subset bootstrap method described by Epskamp et al. (2018). Random subsets of the data, varying in size, were selected, and networks were estimated based on these subsets. For each centrality metric, nodes were ranked in both the full dataset and the subset, and the rank correlation was computed. This process was repeated 1000 times per subset size to obtain an average correlation and a 95% interval encompassing most correlation values. Subset sizes ranged from 95% to 50% of the full sample.

	CROATIA Partnered		CROATIA Non-partnered		GERMANY Partnered		GERMANY Non-partnered	
	avg. corr.	95% interval	avg. corr.	95% interval	avg. corr.	95% interval	avg. corr.	95% interval
**95% sample**	0.756	0.233 - 0.983	0.907	0.733 - 1	0.615	0.141 - 0.950	0.812	0.533 - 0.967
**90% sample**	0.711	0.167 - 0.983	0.892	0.641 - 0.983	0.589	-0.010 - 0.917	0.802	0.517 - 0.959
**85% sample**	0.688	0.141 - 0.975	0.870	0.533 - 0.983	0.575	-0.017 - 0.875	0.787	0.467 - 0.950
**80% sample**	0.648	0.150 - 0.967	0.851	0.483 - 0.983	0.545	-0.133 - 0.875	0.770	0.433 - 0.959
**75% sample**	0.642	0.091 - 0.967	0.824	0.416 - 0.983	0.549	-0.134 - 0.883	0.756	0.408 - 0.933
**70% sample**	0.633	0.050 - 0.967	0.805	0.333 - 0.983	0.548	-0.025 - 0.867	0.751	0.417 - 0.933
**65% sample**	0.607	0.033 - 0.950	0.781	0.358 - 0.983	0.525	-0.083 - 0.850	0.734	0.358 - 0.933
**60% sample**	0.577	-0.033 - 0.950	0.749	0.174 - 0.967	0.536	-0.042 - 0.883	0.696	0.333 - 0.933
**55% sample**	0.553	0.093 - 0.950	0.686	0.017 - 0.967	0.485	-0.175 - 0.867	0.693	0.308 - 0.933
**50% sample**	0.538	-0.083 - 0.933	0.658	0.017 - 0.950	0.487	-0.267 - 0.867	0.686	0.267 - 0.933

Table S4 – Participation coefficient stability by country and relationship status

	CROATIA Partnered		CROATIA Non-partnered		GERMANY Partnered		GERMANY Non-partnered	
	avg. corr.	95% interval	avg. corr.	95% interval	avg. corr.	95% interval	avg. corr.	95% interval
**95% sample**	0.985	0.950 - 1	0.927	0.717 - 1	0.978	0.933 - 1	0.923	0.750 - 1
**90% sample**	0.978	0.933 - 1	0.888	0.525 - 1	0.966	0.883 - 1	0.901	0.400 - 0.983
**85% sample**	0.969	0.908 - 1	0.836	0.325 - 1	0.953	0.867 - 1	0.872	0.367 - 1
**80% sample**	0.960	0.867 - 1	0.810	0.308 - 0.983	0.951	0.867 - 1	0.830	0.333 - 0.983
**75% sample**	0.953	0.850 - 1	0.759	0.191 - 0.967	0.949	0.867 - 1	0.808	0.333 - 0.983
**70% sample**	0.934	0.783 - 1	0.739	0.141 - 0.950	0.938	0.850 - 1	0.779	0.317 - 0.983
**65% sample**	0.917	0.691 - 1	0.696	0.041 - 0.950	0.937	0.850 - 1	0.766	0.308 - 0.983
**60% sample**	0.900	0.683 - 1	0.634	-0.150 - 0.950	0.931	0.833 - 1	0.741	0.283 - 0.983
**55% sample**	0.892	0.667 - 0.983	0.514	-0.150 - 0.950	0.923	0.833 - 0.992	0.697	0.283 - 0.967
**50% sample**	0.866	0.608 - 0.983	0.438	-0.150 - 0.933	0.920	0.833 - 0.983	0.692	0.267 - 0.967
